# Magnetic cryogels as a shape-selective and customizable platform for hyperthermia-mediated drug delivery

**DOI:** 10.1038/s41598-022-13572-9

**Published:** 2022-06-10

**Authors:** Ayomi S. Perera, Richard J. Jackson, Reece M. D. Bristow, Chinyere A. White

**Affiliations:** 1 Department of Chemical and Pharmaceutical Sciences, Kingston University London, Penrhyn Road, Kingston upon Thames, KT1 2EE UK; 2 Department of Mechanical Engineering, University College London, Torrington Place, London, WC1E 7JE UK

**Keywords:** Chemistry, Materials science

## Abstract

Cryogels consisting of polyvinyl alcohol and iron (II, III) oxide magnetic nanoparticles coated with a model drug—acetaminophen, were developed as a tunable platform for thermally triggered drug release, based on shape-selective heat transfer. Two different shapes of cryogels; discs and spherical caps, were formed via adding polymer-nanoparticle-drug mixtures into 3D printed molds, followed by freeze-thawing five times. No additional chemical crosslinking agents were used for gel formation and the iron oxide nanoparticles were coated with acetaminophen using only citric acid as a hydrogen-bonding linker. The two gel shapes displayed varying levels of acetaminophen release within 42–50 °C, which are ideal temperatures for hyperthermia induced drug delivery. The amount and time of drug-release were shown to be tunable by changing the temperature of the medium and the shape of the gels, while keeping all other factors (ex. gel volume, surface area, polymer/nanoparticle concentrations and drug-loading) constant. The discs displayed higher drug release at all temperatures while being particularly effective at lower temperatures (42–46 °C), in contrast to the spherical caps, which were more effective at higher temperatures (48–50 °C). Magnetic hyperthermia-mediated thermal imaging and temperature profiling studies revealed starkly different heat transfer behavior from the two shapes of gels. The disc gels retained their structural integrity up to 51 °C, while the spherical caps were stable up to 59 °C, demonstrating shape-dependent robustness. The highly customizable physicochemical features, facile synthesis, biocompatibility and tunable drug release ability of these cryogels offer potential for their application as a low cost, safe and effective platform for hyperthermia-mediated drug delivery, for external applications such as wound care/muscle repair or internal applications such as melanoma treatment.

## Introduction

Magnetic nanocomposites incorporated into polymeric cryogels is a growing area of interest in materials science due to their superior physicochemical properties^1^, yet, they have not been explored to their fullest potential. Cryogels have the advantages of being biocompatible and cost effective, as they are synthesized by freeze-thawing of aqueous polymer mixtures, and hence, do not contain any reactive, toxic or expensive chemical cross-linking agents^2–5^. They can also be easily combined with magnetic nanoparticles (MNPs) for development of advanced functional materials, particularly for biomedical applications such as hyperthermia^6^ and wound care^7^, thus offering potential for development of sustainable, robust and efficient systems for controlled drug release.

Hyperthermia, a phenomenon broadly defined in biomedicine as elevating tissue temperatures by means of external stimuli beyond normal physiological values, has been used for treatment of cancer^8,9^, Rheumatic diseases (such as fibromyalgia and ankylosing spondylitis)^10^ and immunosuppression in management of pain and inflammation^11–13^. Thus, hyperthermia based treatments are versatile and provide relatively non-invasive approaches to tackle multiple health conditions with scope and potential for further expansion.

Cancer treatment is by far the most-explored area in hyperthermia-mediated drug delivery. It compliments conventional treatment methods including surgery, radiation, immunotherapy or chemotherapy, and can be delivered across either the whole body, or specific regional and local target sites^14,15^. Induction of hyperthermia-level temperatures are achieved via electromagnetic fields (AC), ultra sound, perfusion methods, heat radiation and microwave radiation, depending on the location and depth of the target site^9,14,16,17^. Typical temperature ranges used for hyperthermia for cancer treatment fall into two major categories of temperatures higher than 46 °C and temperatures within 41–46 °C. The former, referred to as thermo-ablation, involves the use of temperatures of > 46 °C with an upper limit of 56 °C, and leads to direct cell death (i.e., necrosis) at target sites^8^. In contrast, the second category utilizes moderate temperatures of 41–46 °C and facilitates apoptotic cell death, and is hence, more widely used.

Nanocomposites consisting of polymer matrices plus metal/metal oxide nanoparticles are gaining prominence in hyperthermia-related research on drug delivery^18^. They aim to combat key drawbacks of conventional methods including: (1) excess and destructive heating of surrounding tissue; (2) under-heating of target areas located deep in the body or covered by hard bone tissue (ex. skull and pelvis); and (3) limited heat penetration from sources such as ultrasound or microwave methods resulting in recurrent tumor growth or incomplete removal^19^. Currently, advanced nanocomposite systems such as fibres^20^, hydrogels^21,22^, sol–gels^23^ and liposomes^24,25^, that can be directly delivered or injected into target sites and carry drug payloads mounted on nanoparticles, are being investigated as potential methods to reduce side effects and enhance hyperthermia efficiency. One class of such materials gaining rapid prominence are magnetic nanocomposites that contain superparamagnetic nanoparticles (i.e., NPs that display enhanced magnetic properties in the presence of external magnetic fields) such as iron oxides^8,9,26^.

The use of MNPs are advantageous as they can be readily triggered by external magnetic fields or radiation to convert dissipated magnetic energy into heat, and are hence, an effective source of inducing hyperthermia^9^. Moreover, MNP-based delivery systems have desirable features such as: (1) they are easily absorbed into cancer cells due to their small sizes; (2) they can be delivered via multiple routes; (3) they can be functionalized with various drugs or target-specific binding agents to increase selectivity and efficiency of treatment; (4) they have high heating efficiency due to magnetic properties, hence, less energy required via external stimuli etc. Several clinical studies have indeed shown promise^27,28^, however, despite above incentives and decades of intense research, MNP-based materials are yet to enter into real world, day-to-day applications in healthcare. Nonetheless, their beneficial features offer the possibility to develop advanced and multidimensional approaches to non-invasive and precise drug delivery. The importance of research into novel materials for magnetic hyperthermia induced drug delivery, targeting less explored external applications such as wound care, muscle repair etc. is therefore paramount.

Wound healing in particular, is emerging as a promising area for investigation, with hyperthermic heating of magnetic nanoparticles acting as effective broad spectrum antimicrobials^29,30^, as well as providing various cues for neural regeneration^31,32^. At lower frequencies (< 100 Hz) magnetic fields can also modulate mechanosensitive ion channels in cells^33^. General heating in therapeutic ranges achievable with these approaches have been shown to act as gene expression triggers^34^, while systems containing MNPs in conjugation with enzymes^35^ and synthetic vesicles^36^ have shown promise in enhanced wound healing. Hence, it is likely that a multimodal approach that involves heat-mediated drug release together with cryogelation would have a number of useful applications in regenerative medicine.

In a previous publication we demonstrated that PVA-MNP nanofibers, synthesized by pressurized gyration can be used to remotely control drug release under magnetic actuation^20^. Building on this technology, the current study was aimed at developing a customizable magnetic nanocomposite hydrogel platform, which is biocompatible and low-cost, using polyvinyl alcohol (PVA) cryogels incorporated with acetaminophen(Ac)-coated iron (II, III) oxide (Fe_3_O_4_) nanoparticles, for thermally triggered, controlled drug release. Results detailed herein reveal that the material can be readily tuned to release different amounts of the drug, controlled exclusively via external temperature and shape of the cryogels. The influence and mechanisms of heat transfer within differently shaped cryogels are also discussed.

## Materials and methods

Iron (II, III) oxide nanopowder (15–20 nm) was sourced from US Research Nanomaterials, Inc. All other chemicals reagents were sourced from Sigma Aldrich and were used without further modification.

### Functionalization of iron (II, III) oxide MNPs

#### Citric acid coating of MNPs (MNP-CA)

25 g of citric acid was added to 150 ml of deionized water and heated to 90 °C in an Erlenmeyer flask. Next, 2.00 g of Iron (II, III) oxide nanopowder (US Research Nanomaterials, Inc., 15–20 nm) was added to the solution. Then the solution was stirred magnetically for 2 h at 90 °C. Afterwards, the supernatant was removed while a neodymium magnet was placed at the bottom of the Erlenmeyer flask to prevent the solid nanoparticles from being removed. The nanoparticles thus collected were washed using distilled water twice and dried in oven at 80 °C for 2 h (adapted from Nigam et al.^37^).

#### Incorporation of acetaminophen/paracetamol into MNP-CAs (MNP-CA-Ac)

2 g of MNP-CA were added to 50 ml of deionized water containing 100 mg of acetaminophen (2 mg/ml) in an Erlenmeyer flask. The solution was then heated to 40 °C and stirred mechanically for 2 h. Next, the solution supernatant was decanted by holding a neodymium magnet underneath the Erlenmeyer flask to hold back the MNPs from being decanted. The MNPs collected were then washed once with distilled water and oven-dried at 80 °C, for 2 h. The weight of the dried coated MNPs recovered was 1.4 g, indicating a 30% weight loss of MNPs due to corrosion by excess aqueous citric acid throughout the procedure.

### Synthesis of the polymer-magnetic hydrogels

In order to synthesize poly vinyl alcohol (PVA) hydrogels, first a 5% PVA solution was formed by adding 5 g of PVA to 100 ml of deionized water, at 90 °C with magnetic stirring for 2 h^2^. The solution obtained was stored at 4 °C in the fridge for later use and was stable up to 1 month. Next, the drug-coated MNPs were incorporated to the PVA solution as follows: 20 ml of the PVA solution was mixed with MNP-CA-Ac particles by 5% (w/w%), and mechanically stirred at room temperature until a homogenous mixture was obtained. Then, 2 ml portions of this mixture was syringed into 3D printed disc-shaped molds, of 2.2 cm diameter and 1.5 cm depth, and placed in a –20 °C freezer for 1 h, which was immediately followed by thawing at room temperature for another 1 h. This process was continued for a further 4 cycles of freezing and thawing, resulting in 5 freeze–thaw cycles in total, which led to formation of magnetic hydrogels with a stable gel structure with 0.5 cm height.

**Figure 1:**
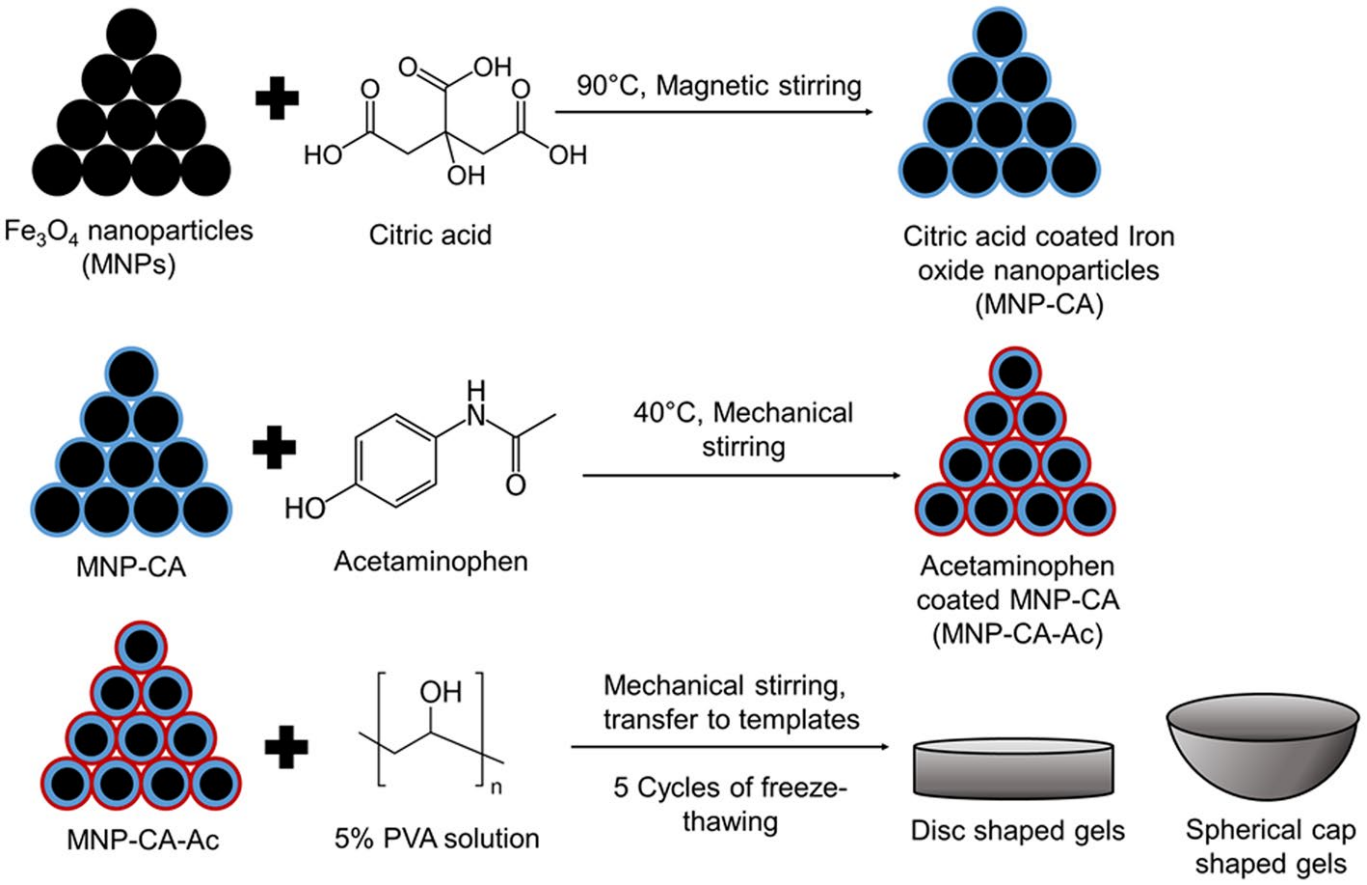
Schematic representation of the synthesis of disc and spherical cap shaped PVA-MNP-CA-Ac cryogels.

A second set of gels were prepared by syringing 2 ml of the PVA-MNP-CA-Ac mixture (prepared as described above) into 3D printed spherical cap-shaped molds of 2.5 cm diameter and 2.5 cm depth, followed by 5 cycles of freeze-thawing. This led to the formation of stable gels with 0.7 cm height (Fig. 1).

For investigation of the effect of additional hydrogen bonding on magnetic hyperthermia induced heat transfer, gels consisting of MNPs with only citric acid coating without the drug (i.e., PVA-MNP-CA) were also fabricated. This was done by mixing 5% PVA solution with 5% (w/w) MNP-CA, followed by syringing 2 ml of PVA-MNP-CA slurry to disc and spherical cap shaped molds and subsequent freeze-thawing, according to similar procedures as discussed above (see “Citric acid coating of MNPs (MNP-CA)", "Synthesis of the polymer-magnetic hydrogels” sections). The drug loading efficiency of the gels was found to be 4.88% (w/w) for acetaminophen/MNP-CA (48.8 mg of acetaminophen loaded onto 1 g of citric acid coated MNPs), measured via UV–Vis based concentration from a fully dissolved gel in a 25% solution of IMS:0.1 M HCl.

### Characterization

Chemical structure of the coated and non-coated MNPs as well as MNP-incorporated hydrogels, were characterized with Fourier Transform Infra-Red Spectroscopy (FTIR) and Thermogravimetric Analysis (TGA). FTIR was performed with a Thermofisher iS5 spectrometer with iD7 ATR accessory, to detect the presence of citric acid and acetaminophen in the coated MNPs and was compared against the uncoated MNPs and commercial citric acid and acetaminophen. Dynamic light scattering (DLS) based particle sizing and zeta potential (ZP) experiments were conducted using a Malvern Zetasizer instrument. TGA was performed on a Mettler Toledo instrument, in an aluminium crucible, where the coated MNPs were analysed using a 5 °C/min ramp rate from room temperature to 500 °C and uncoated MNPs, commercial citric acid and acetaminophen were analyzed with a 20 °C/min rate up to 500 °C, with the data plotted using Mettler—STARe Evaluation software. Morphology of the hydrogels were characterized by SEM/EDX to observe the gel pore structure and determine the iron oxide nanoparticle distribution within the hydrogels. Prior to analysis, the hydrogels were freeze-dried to remove water using BenchTop Pro VirTis, followed by gold coating. Images were taken under a ZEISS EVO 50 scanning electron microscope, with the related X-ray maps being produced with the associated OXFORD INSTRUMENTS INCA X-ray microanalysis suite. Drug release was characterized via UV–Vis analysis using a Magellan infinite m200 pro plater reader.

### Thermally triggered drug release studies

The disc-shaped and spherical cap-shaped gels were subject to thermally triggered drug release experiments. For each experiment, the rim of a 100 ml glass beaker containing a magnetic stirrer was fixed with a stage made from plastic mesh using tape. Then, 40 ml of 25% ethanol in deionized water solution was added to the beaker, while ensuring the part of the mesh stage was immersed in this solution (ESI, Fig. S1). This system was then submerged in a water bath with a set temperature, and the temperature of both systems was allowed to homogenize, under magnetic stirring. Next, the hydrogel was added onto the mesh stage, while ensuring it is completely immersed in the ethanol solution. In order to determine the concentration of acetaminophen released from the gel, 300 μl samples were collected from the beaker 5 min intervals up to 65 min. The extracted solvent was replaced with another 300 μl of fresh solvent, each time to ensure a constant volume throughout the experiment. This experiment was conducted at a range of temperatures, beginning with a control experiment at room temperature (i.e., 22 °C), followed by at 40, 42, 44, 46, 48 and 50 °C. Each of the above experiments was repeated twice for accuracy, and their standard deviation was used as experimental error. The collected samples were stored in Eppendorf tubes and later analyzed via UV–Vis absorbance using a Magellan infinite m200 pro plater reader. Absorbance values were converted to concentration via a standard calibration curve for acetaminophen.

### Hyperthermia imaging studies

The hyperthermia set up consisted of a copper coil and a Hikvision ds-2tp21b-6avf/w thermal imaging camera for imaging the temperature of both disc-shaped hydrogels and hemispherical hydrogels. The coil was heated under 19.95 V with a 14 Amps (AC) current at a frequency of 0.812 MHz. The gels were kept in a Teflon container at the center of the coil and subject to heating while thermal images were taken every 10 s for 20 min. Two different types of magnetic hydrogels were tested for each shape; one with MNP with citric acid + acetaminophen coating (MNP-CA-Ac), and the other with MNPs with only citric acid coating (MNP-CA). As a control, 5% PVA gels in both disc and cap shapes, without any MNPs were also studied. After heating for 20 min the current was switched off and the gels left to cool for a further minute, with images taken every 10 s up to the minute. The images thus obtained were interpreted using iVMS 4800 thermal imaging software in order to determine the rate of heating of the hydrogels over time.

## Results and discussion

### Chemical characterization of coated nanoparticles

**Figure 2:**
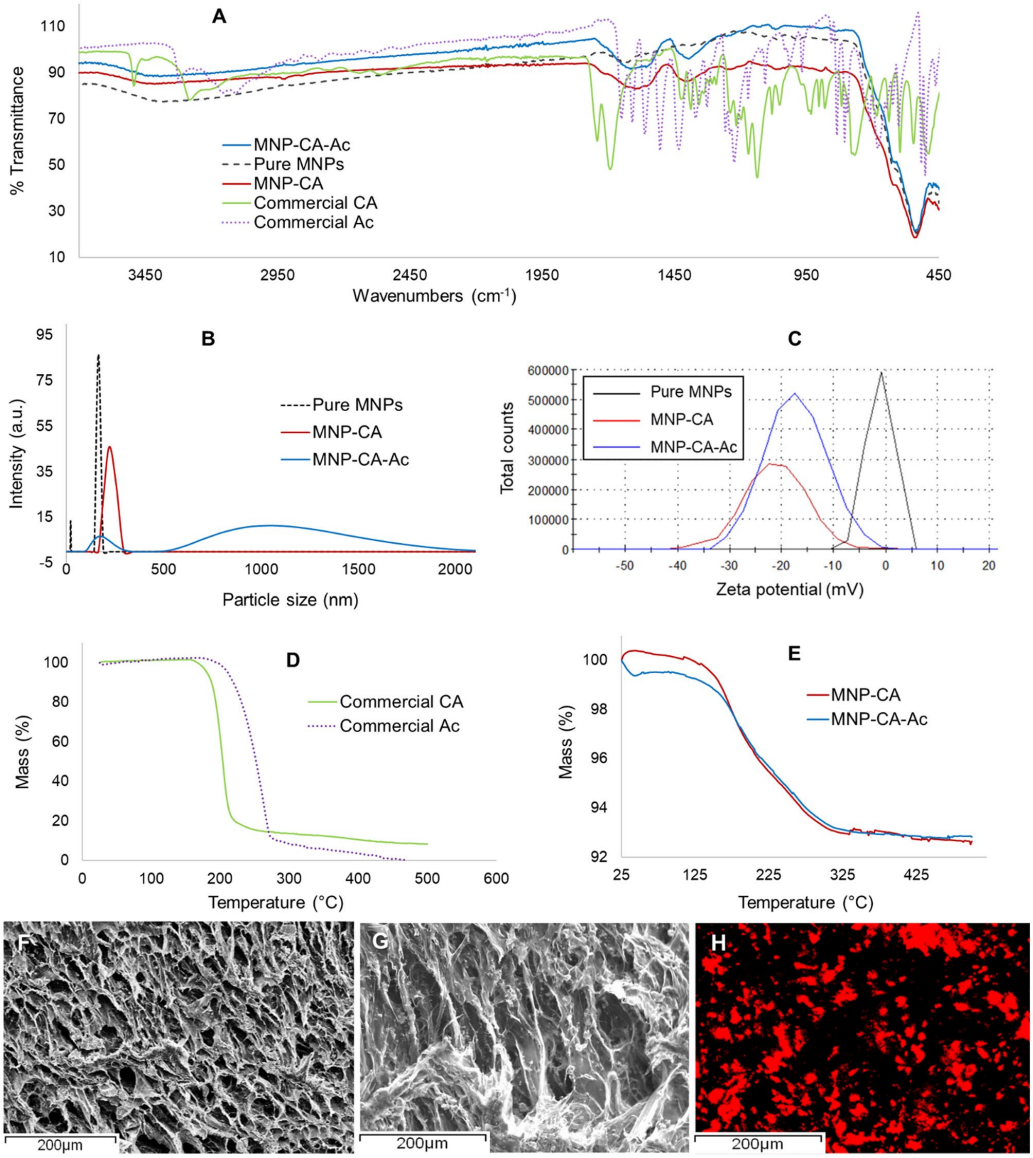
Characterization of PVA-MNP hydrogels. (**A**) FTIR spectra of all samples. (**B**) DLS data for pure MNPs, MNP-CA and MNP-CA-Ac samples indicating differences in particles sizes. (**C**) Zeta potential data for pure MNPs, MNP-CA and MNP-CA-Ac samples indicating differences in surface charges. (**D**) TGA data for commercial citric and acetaminophen. (**E**) TGA data for MNP-CA and MNP-CA-Ac samples. (**F**, **G**) SEM images of freeze-dried drug coated PVA-MNP hydrogels. (**H**) EDX data for SEM image G, where drug coated MNP distribution is depicted in red, indicating a homogeneous distribution of the MNP-CA-Ac system within the hydrogel.

Chemical composition of the cryogels were analyzed using FTIR, TGA and EDX. FTIR spectrum for pure Fe_3_O_4_ powder (pure MNPs) showed a strong absorption at 538 cm^−1^ indicating Fe–O vibration (Fig. 2A). Typical literature value for this peak is around 580 cm^−1^^38,39^, however, this higher wavelength shift could be due to the polar environment of the KBr pellet^40^, for which our sample was not exposed. Other prominent bands from this sample included a broad peak around 3350 cm^−1^ and another at 1630 cm^−1^ corresponding to surface hydroxyl groups^38,40^. Citric acid coated sample showed broad peak at 3400 cm^−1^ corresponding to hydrogen bonding of OH groups. The two peaks at 1597 cm^−1^ and 1412 cm^−1^ indicative of C=O symmetric stretch and asymmetric stretch respectively, resulting from the carboxylic acid groups of citric acid^41^. Corresponding peaks from the commercial citric acid powder were found at 1741 cm^−1^, 1692 cm^−1^ (C=O symmetric stretch) and 1424 cm^−1^ (asymmetric stretch). The shift to lower wavelengths found on the MNP-CA sample indicate chemisorption of citric acid on the Fe_3_O_4_ surface via interaction of the –COOH groups of CA with the surface Fe-OH groups on MNPs, thus rendering partial single bond character to the C=O bond leading to lower shift of its wavelength^42^. This interaction (i.e., coating of citric acid on MNP surface) plays a key role in preventing particle aggregation of MNPs in solution, thus ensuring their homogeneous mixing into the PVA mixture.

FTIR spectrum of the commercial acetaminophen sample indicated a peak at 3319.64 cm^−1^, characteristic of the O–H stretching vibration, which was shifted to 3447 cm^−1^ in the MNP-CA-Ac sample as discussed above. CH_3_ and aromatic C–H stretching in the commercial sample appeared at 2792.84 cm^−1^ and 3107.64 cm^−1^ respectively, while the C=O stretching of the amide was discernible at 1650.34 cm^−1^ and the peak at 1327.07 cm^−1^ is likely due to the C–N stretching of the aryl amide. The C–O stretch of the tertiary alcohol appeared at 1171.28 cm^−1^ and the C–N stretching from the amide was seen at 1107.24 cm^−1^. Corresponding peaks were identified in the MNP-CA-Ac sample at 1651, 1392, 1123 and 1053 cm−^−1^, respectively, with the shifts in the latter three peaks being considered to be due to hydrogen bonding of the amide and tertiary alcohol of acetaminophen with citric acid.

DLS data (Fig. 2B) shows distinct differences between particle size aggregation between pure MNPs, MNP-CA and MNP-CA-Ac samples dispersed in DI water. Pure MNPs have peak sizes of 21 nm and 164 nm, with MNP-CA having a peak size of 220 nm and MNP-CA-Ac have a minor peak at 164 nm and a shoulder from 500 to 2300 nm. Likewise, zeta potential data (Fig. 2C) indicate differences in surface charge of the particles, with a peak potential of 0.06 mV for pure MNPs and − 21.1 mV for MNP-CA. The negative charge on the latter is due to free carboxylic groups and indicates effective coating and dispersion via charge stabilization^43^. The MNP-CA-Ac showed a zeta potential of − 17.2 mV. The above increase in overall surface charge indicated the bonding between acetaminophen and the free carboxylic groups of citric acid via electrostatic interactions^37^.

TGA data for the commercial citric acid powder showed one mass loss event at 197 °C that accounted for 88% of weight loss (Fig. 2D). In contrast MNP-CA sample showed two mass loss events at 175 °C and 272 °C, which accounted for 5.4% and 2.3% loss of weight respectively (Fig. 2E), indicating the desorption of acid molecules from the MNP surface. The two events were more discernible when examining the derivative of the TGA curve (ESI Fig. S2), and could be a result of bilayer formation of citric acid on the MNP surface, as previously reported^44–46^. Here, an external layer of citric acid, hydrogen bonded to an inner layer desorbs sooner and at a lower temperature, followed by the inner layer, which is hydrogen bonded to the MNP surface and desorbs later at an elevated temperature. The TGA of commercial acetaminophen power showed only one mass loss event at 245 °C, accounting for 94% of weight loss (Fig. 2C). However, the drug-coated MNP-CA-Ac sample showed two TGA mass loss events at 176 °C and 264 °C, which were 3.6% and 2.8% of weight loss respectively (Fig. 2D). Overall, the TGA curves for both MNP-CA and MNP-CA-Ac samples appear very similar, and are hence, effectively non-distinguishable from each other. Moreover, the total weight loss from the drug-containing sample was lower than that of the MNP-CA sample. These complications were likely a result of bilayer formation of citric acid that occurred on the MNP-CA sample as discussed above, thus preventing an accurate weight % determination for acetaminophen coated onto the MNP system via TGA. The slight weight increase seen in MNP-CA sample at lower temperature is attributed to instrument stabilization error which we observed to occur at minor weight changes of > 1%.

The SEM images for the PVA-MNP cryogels indicated a highly porous gel structure (Fig. 2F, G). EDX data with Fe labelling indicated that the MNPs were uniformly distributed throughout the gel (Fig. 2H), verifying that the citric acid coating was effective in homogeneous distribution of MNPs within the gel matrix, while also acting as a hydrogen-bonding anchor for acetaminophen.

### Thermally-triggered drug release studies

#### Developing a customizable drug release platform

The cryogels are formed through 5 cycles of freeze-thawing at − 20/room temperature °C. During this process promotes the aggregation of polymers or entanglement when the ice crystals that form during freezing melts down when thawed, dragging along polymer units with them. Hence, successive freeze-thawing can increase the aggregation of free polymer chains, forcing more residual free chains into larger, bonded structures^47^. Accordingly, the strength of the gels can be influenced by changing the freezing temperature and number of freeze–thaw cycles. A 5% PVA concentration was chosen to achieve a gel stiffness, which allows for drug release via thermal trigger and magnetic hyperthermia. We also considered a desirable gel robustness suited for wound care applications while still being sufficiently flexible to be compatible with body tissue.

A key objective of this study was to investigate the feasibility of releasing controlled amounts of a drug from the PVA-MNP cryogels via external temperature stimuli. This was demonstrated successfully by release of controlled amounts of acetaminophen, triggered by changes to temperature in the surrounding solvent-medium. The medium for all drug release experiments was chosen to be 25% ethanol/DI water, based on investigation of various ethanol: DI water ratios, in order to minimize the experimental errors arising from gel shrinkage/expansion, which allows for more precise investigation of drug release concentrations in this proof-of-concept study (Discussed in ESI, Table S1).

Acetaminophen was chosen as a model drug for this study due to its high solubility in ethanol and water^48^, its characteristic UV–Vis absorption^49^, and its ability to form hydrogen bonds with citric acid. It typically display a strong UV absorption peak at 243 nm and a broad shoulder at around 290 nm, in water^50^. In 25% ethanol/DI water medium, these peaks were observed at 248 and 285 nm, respectively (ESI, Fig. S3). A calibration curve for acetaminophen was obtained using standard solutions and was used to determine the concentration of its quantities released during the thermally triggered acetaminophen release experiments (ESI, Fig. S4).

**Figure 3:**
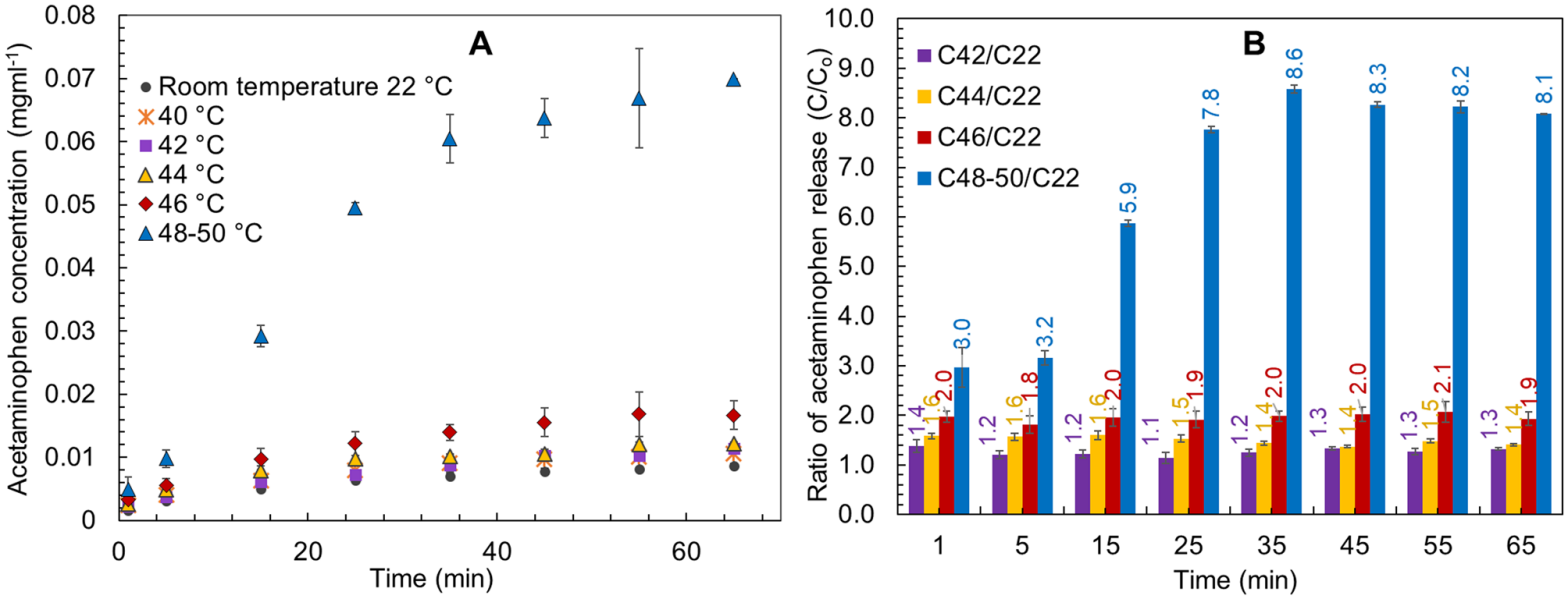
Results for acetaminophen (Ac) release with time from disc shaped gels. (**A**) Concentration of Ac released for disc shaped gels at 22 (r.t.) 40, 42, 44, 46 and 48–50 °C. (**B**) Ratio of Ac released at all temperatures compared to room temperature (i.e., (C40/C22 = concentration at 40 °C/concentration at 22 °C etc.).

The disc shaped cryogels containing MNP-CA-Ac were analyzed for drug release at room temperature (i.e., 22 °C), followed by 40, 42, 44, 46 °C (± 1 °C) (Fig. 3A). Experiments were also conducted at 48 and 50 °C in order to determine the threshold of gel decomposition and its effects on drug release. However, at these temperatures the disc gels started to melt, releasing considerable amounts of drug-loaded MNPs into the ethanol/water medium, which caused ± 2 °C fluctuations in temperature in the solution. Hence, experiments conducted at 48 and 50 °C were averaged and presented as 48–50 °C in the drug release graphs in Fig. 3. Change of acetaminophen concentration vs time, at each temperature was monitored by collecting samples at every 5 min up to 65 min in total.

The curves display typical biphasic release behavior of a hydrophilic drug with initial burst release followed by saturation with time (i.e., zero order release)^51^. This effect was mostly pronounced in the 48–50 °C temperature range. Amounts of acetaminophen released did not differ significantly between 22 (r. t.), 40 °C runs, but indicated a slight increases at 42 and 44 °C (Fig. 3A). The 46 °C run showed noticeably higher amounts of drugs released, while the 48–50 °C runs showed the most significant increase in drug release. In order to quantitatively ascertain the effect of increasing temperature on drug release from the PVA-MNP cryogels, the ratio of acetaminophen concentrations released between temperatures ≥ 40 °C and room temperature were plotted against time (Fig. 3B). This indicated that at 42 and 44 °C, 1.25 and 1.5 times more drugs are released on average throughout the 65 min experiment respectively, with only minor fluctuations, followed by a 2.0 times increase at 46 °C. At 48–50 °C however, this effect increased dramatically. There was a gradual increase in the ratio up to 35 min with an 8.6 times of maximum release, which then plateaued during 45–65 min to a final value of 8.1. These results indicate that changing the temperature in a controlled manner lead to precise and tunable amounts of drugs being released from the cryogels with time, demonstrating the feasibility of a customizable drug-release approach.

**Figure 4:**
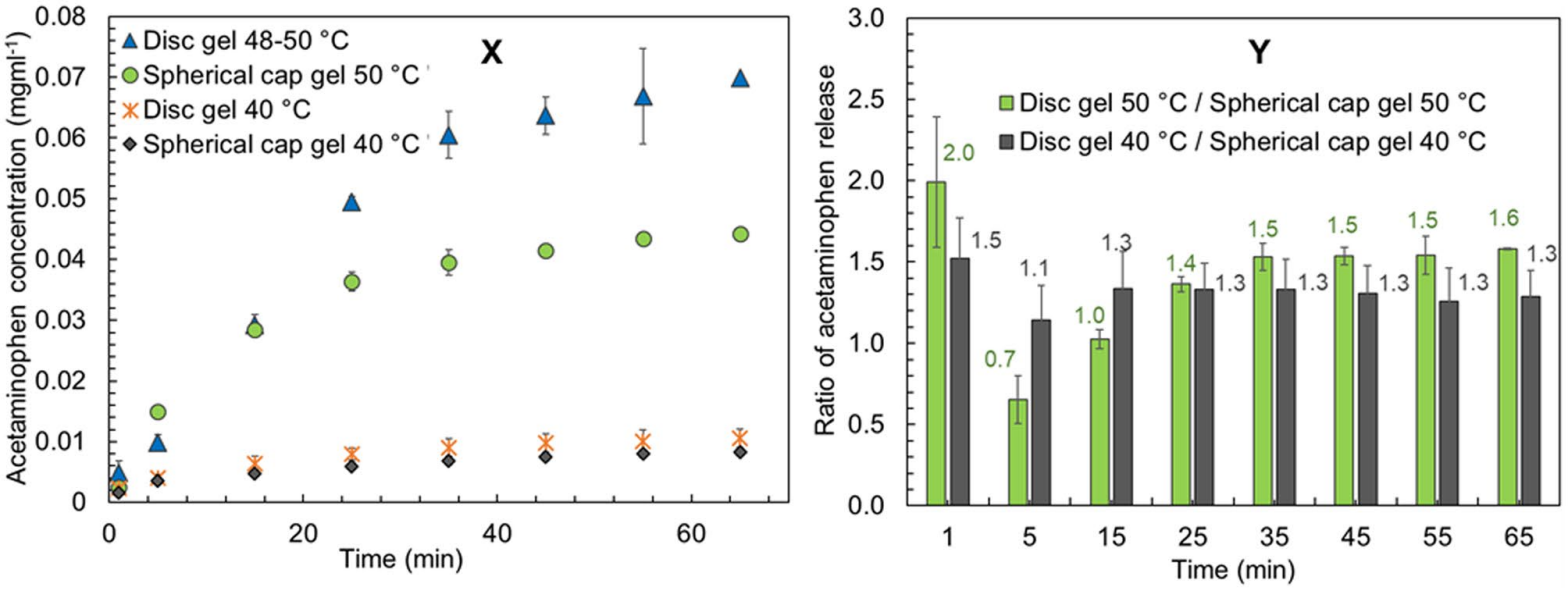
Results for acetaminophen (Ac) release with time from disc shaped vs spherical cap shaped gels. (**X**) Comparison of Ac release concentrations from disc and spherical cap shaped gels at 40 and 50 °C. (**Y**) Ratio of Ac released from disc and spherical cap shaped gels at 40 and 50 °C (note: for disc shaped gels an average of 48–50 °C results were used for graph **Y**).

**Figure 5:**
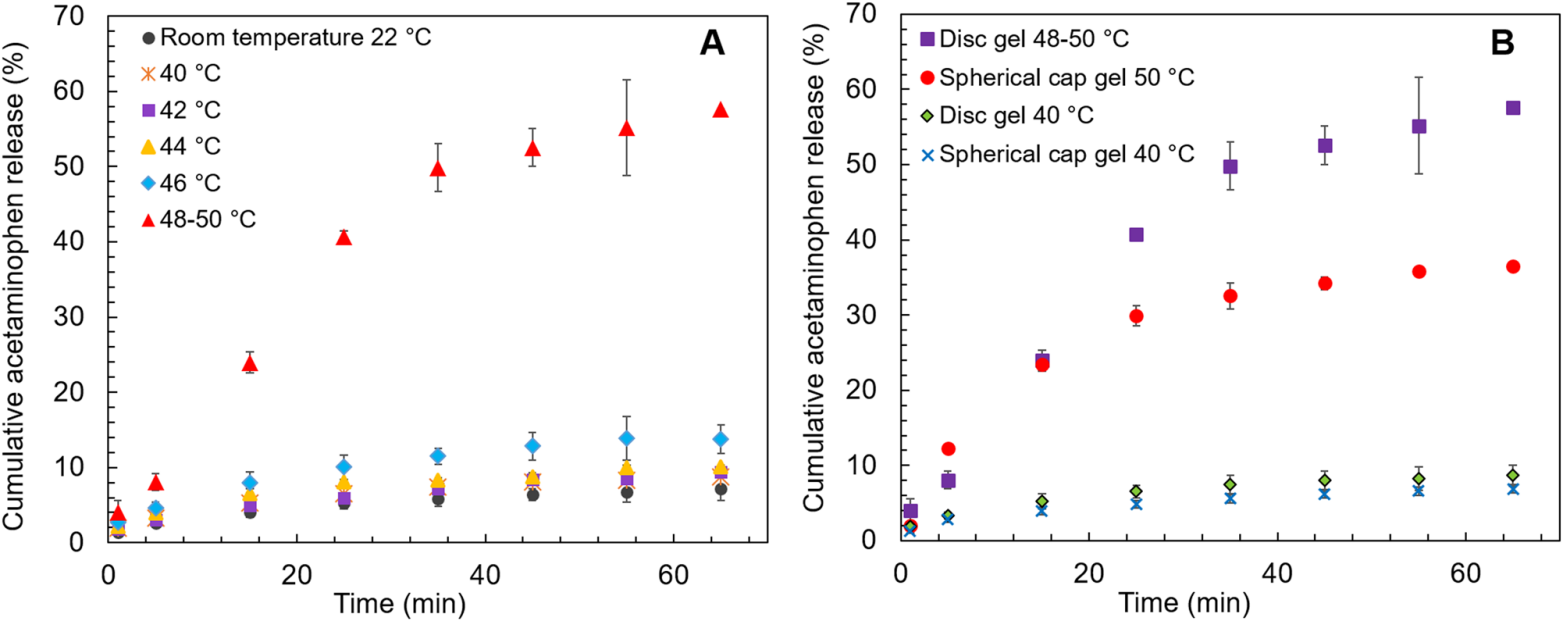
Cumulative acetaminophen release % with time from disc shaped (**A**) and disc vs spherical cap shaped (**B**) gels.

Another key objective of the study was to demonstrate that tunable drug release could be achieved by only changing the shape of the cryogel platform while all other conditions are kept constant. Building on this premise, we explored drug release behavior of spherical cap cryogels at three key temperatures of 22 (r.t.), 40 and 50 °C, selected based on the release trends from the disc shaped gels, and compared against them (Fig. 4). At r.t. the drug release behavior from spherical cap gels was non-distinguishable from the r.t. release of disc shaped gels. At 40 °C there was a marginal difference, where the spherical caps showed slightly less release than the discs (Fig. 4X). At 50 °C however, the drug release from spherical caps increased significantly compared to r.t. and the gels were visibly starting to melt, echoing the behavior of the discs. A notable difference between the shapes however, was that spherical caps released considerably less drug compared to the discs at 50 °C. Examination of ratios of release concentrations confirm the fact that at 40 and 50 °C, the amount of drugs released is 1.3 times and 1.6 times higher respectively, from the discs compared to the spherical caps after 65 min (Fig. 4Y). Cumulative acetaminophen release from disc gels at 65 min were 57.6%, 13.8%, 10.1%, 9.4%, 8.7%, 7.1% and 7.1% at 48–50 °C, 46 °C, 44 °C, 42 °C, 40 °C and r.t. 22 °C respectively (Fig. 5A), further evidencing temperature based customizability of the system. Similarly, cumulative release from spherical cap gels at 65 min were 36.5% and 6.8% at 40 °C and 50 °C (Fig. 5B), depicting the shape-based differences in drug release.

**Figure 6:**
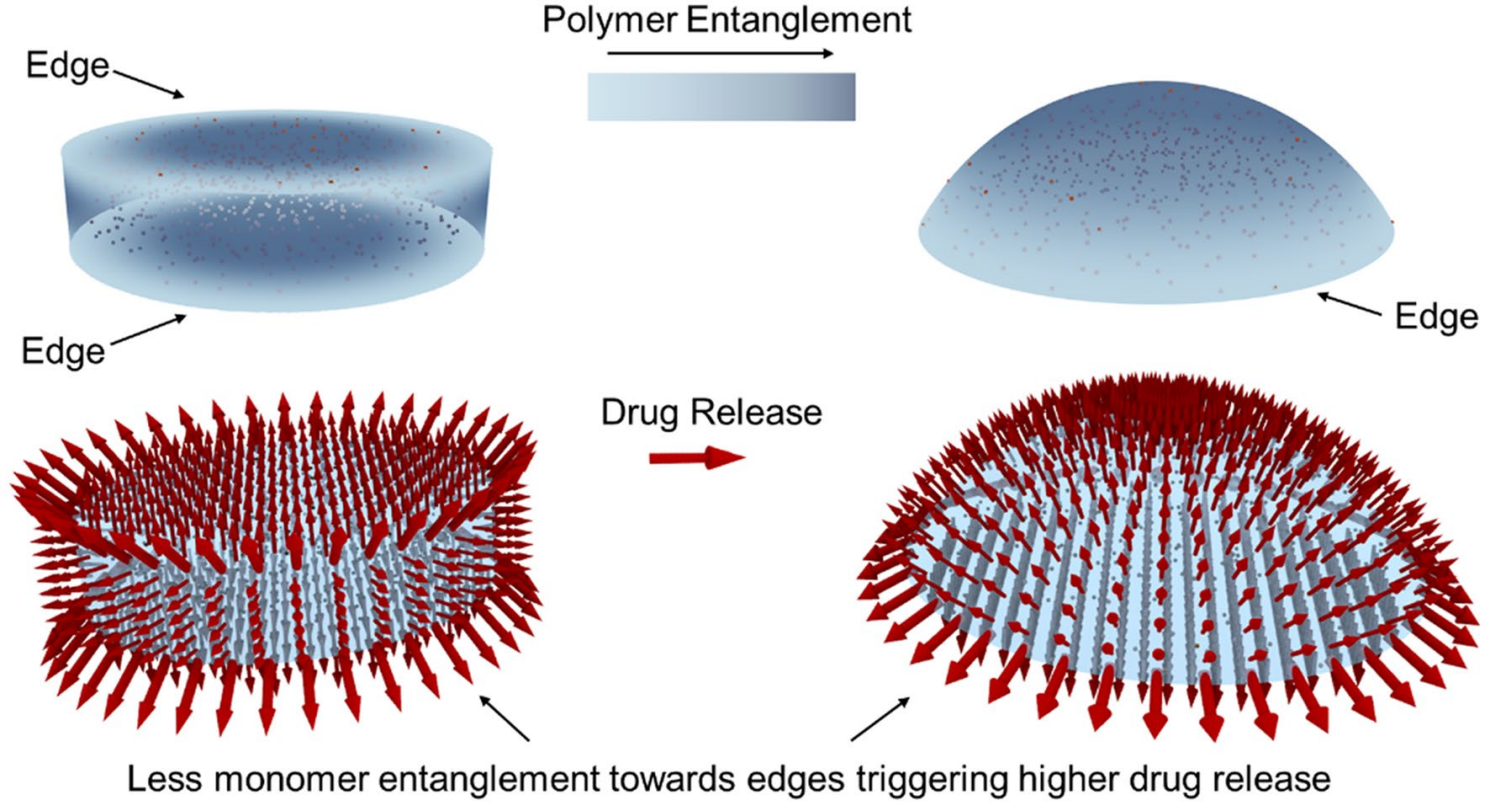
Schematic representation of the proposed mechanism of drug release from PVA-MNP cryogels. Top left: disc shaped gels with two edges, top right: spherical cap shaped gels with one edge. Polymer entanglement is less close to the edges of the gels as indicated by the lighter color and high towards the centers as indicated by the darker color. Bottom left and right: arrows depicting varying levels of drug release from disc and spherical cap gels. Larger arrows indicate higher drug release close to the edges and the smaller arrows indicate less release towards gel centers.

These results reinforce the validity of the shape-selective customizability hypothesis, where gel shape appear to be a clear factor in controlling drug release, with changing temperature of the medium. This shape-based tunable nature offers potential to precisely and conveniently, control the amount of drugs released from the cryogel platform. Interestingly, the surface areas of both shapes are not appreciably different and are within > 0.01% (see ESI Section 1). However, there are significant differences in the shape of the gels, and the occurrence of edges, as the discs have twice as many edges compared to the spherical caps (Fig. 6). This seem to be a critical factor in drug release as evidenced by the increased acetaminophen release from the discs at 40 and 50 °C, compared to the spherical caps.

Although some reporting of such shape-dependent heating behavior of hydrogels exist^52,53^, a comprehensive analysis of polymer-MNP systems considering edge-selective heat transfer under hyperthermia while all other parameters remain constant (including gel volume, surface area, polymer/MNP concentrations and drug-loading), are yet to be reported. Our data indicate that the available edges in each shape is a key factor in influencing hyperthermia-based drug release. This cryogel system has no additional cross-linking agents and the polymer chains are interlinked only via entanglement during freeze-thawing^54^. It can be hypothesized that the edges of the cryogels may contain more loosely entangled polymer chains with less hydrogen bonding within the polymer-composite matrix compared to the center. When the gel temperature is increased, these polymer chains will start to become untangled. Moreover, hydrogen bonds between within the PVA-MNP-CA-Ac system will be disrupted, resulting in higher drug release. As the cryogel approaches melting temperature this effect will be more pronounced, leading to an exponential increase of the rate of drug release around ~ 50 °C, as evidenced by our results (see Figs. 3, 4, 5). This can explain why the discs which have two circular edges compared to the spherical caps which only have one such edge, display significantly higher amounts of drug release.

### Magnetic hyperthermia experiments: understanding shape-selective heat transfer in magnetic cryogels

In order to visualize and monitor magnetically induced heat transfer interactions within the gels, magnetic hyperthermia and concurrent thermal imaging were conducted with the two shapes of PVA-MNP cryogels, with and without drug coating on MNPs (i.e., MNP-CA and MNP-CA-Ac respectively) (Fig. 6). The former category only had citric acid coating to achieve uniform distribution of MNPs within the gel and the latter included an additional acetaminophen coating. The gels were placed in a Teflon container, which was then inserted in a copper coil, followed by exposing to an electromagnetic field via AC current application (ESI Fig. S5), which generated a frequency of 0.812 MHz and a field strength of 11.2 × 10^9^ A/m (ESI Section 2). The field at the center of the coil used within this study is of the order of agreed safety limits for internal tumor treatment times of around an hour (5 × 10^9^ A/m)^55^ but around double, as our system ran at full power for this initial proof-of-concept study. Likely clinical applications in external topical or wound care targeted drug release would be less demanding in heat dissipation. Optimization of materials, shape and process parameters could be targeted towards lowering this value further.

**Figure 7:**
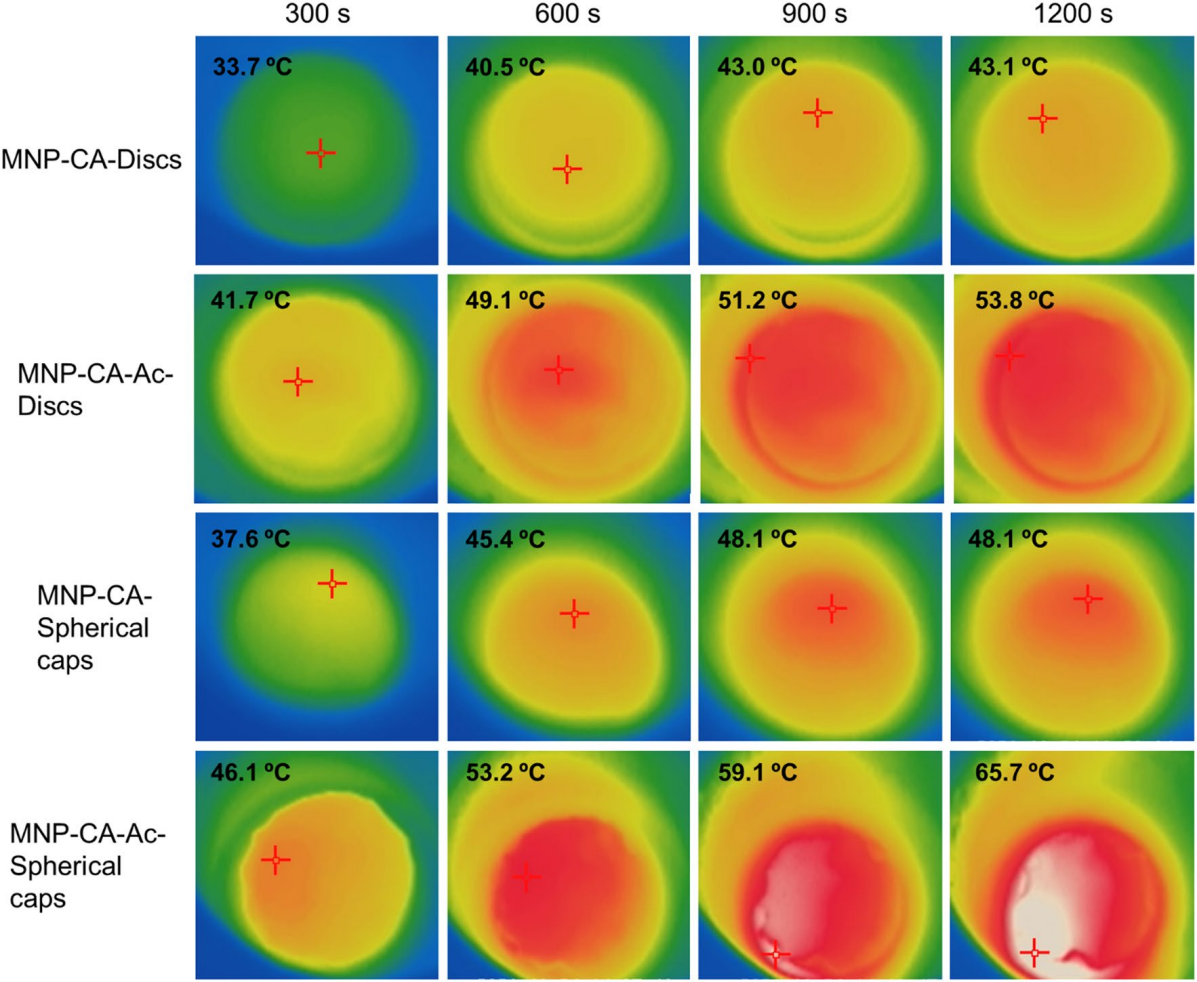
Thermal images indicating magnetic hyperthermia mediated temperature increase of disc-shaped and spherical cap shaped gels with and without acetaminophen coating (MNP-CA and MNP-CA-Ac respectively) over time. The hyperthermia set up consisted of a copper coil and a Hikvision ds-2tp21b thermal imaging camera. The coil was heated under 19.95 V with a 14 A (AC) current at a frequency of 0.816 MHz. Gels were kept in the center of the coil on a Teflon container and subject to heating while thermal images were taken every 10 s for 20 min.

Thermal imaging revealed gradual heating of the gels under magnetic hyperthermia, starting from their centers and spreading to the edges (Fig. 7). This is compatible with the polymer entanglement being more pronounced at the gel centers, compared to the edges as discussed above, thus aiding heat transfer via increased conduction pathways^56^. Unlike in solution-based systems, there is less risk of isolated transient ‘hotspots’ forming within these gels due to strong immobilization of MNPs within a heavily hydrogen bonded system in the cryogels. We observed a continuous heating gradient across all gels over time. Continuation of magnetic hyperthermia eventually led to melting of both disc and spherical cap shaped gels. Melting times were dependent on shape and whether or not the MNPs were drug-coated.

**Figure 8:**
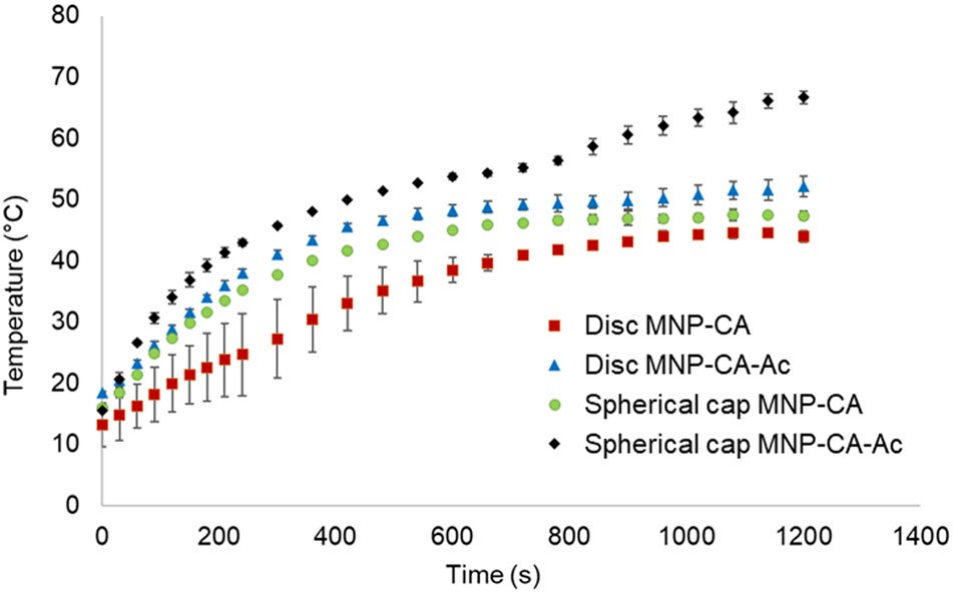
Heat transfer behavior of disc and spherical cap PVA-MNP cryogels, with and without acetaminophen coating against time.

Overall, the spherical cap gels showed increased heat transfer compared to the discs, both with and without the acetaminophen coating (Figs. 7, 8). Additionally, both shapes showed significantly higher heat transfer when acetaminophen was incorporated, compared to without it, indicating that the extra hydrogen bonding and/or electrostatic attractions within the gel matrix emanating from the drug molecules enhances heat transfer. In disc shaped gels without drug (Disc-MNP-CA), the temperature increased gradually and plateaued at 43 °C by 900 s, before the end of the 1200 s experimental time, while the discs with drug (Disc-MNP-CA-Ac) heated much more sharply and reached 53.8 °C during the same time (Fig. 8). The spherical cap gels without drug (Spherical cap-MNP-CA) also reached a stable temperature of 48.1 °C by 900 s and plateaued, while those with drug coating (Spherical cap-MNP-CA-Ac) showed a stark temperature increase, reaching a maximum of 65.7 °C by the same time. Control experiments with 5% PVA gels of both shapes that did not contain any MNPs, showed no notable temperature increase beyond room temperature (ESI Fig. S6), confirming that the temperature increase in magnetic gels are caused by the magnetic properties in MNPs.

These intriguing changes in magnetically induced heat transfer are notably linked to gel shape and by extension to changes polymer entanglement and hydrogen bonding within the PVA-MNP matrix. This is in agreement with previous reports that indicate that thermal conductivity in polymeric hydrogels are enhanced by increased crosslinking, due to enhanced conduction pathways^56^. Studies that look into Brownian motion and Néel relaxation behavior of MNPs immobilized within gel matrices indicate that their heating efficiency decreases with increased degree of immobilization^57^. This was confirmed by us via hyperthermia mediated heat transfer of PVA-MNP slurries (i.e., without any cross linking via gel formation), which showed significantly higher temperature increases regardless of drug coating, compared to the cross-linked cryogels (ESI Fig. S7). We did not however, find any previous reporting on the shape selective heat transfer behavior of MNP-cryogel systems described herein, where concentrations of polymer, MNP, drug-loading and surface area together with magnetic hyperthermia conditions are all kept constant. In previous reports, the abovementioned factors are varied, and proved to have a direct impact on heat transfer within magnetic-hydrogel systems^58,59^. The enhanced heat transfer of the drug-coated gels in our study together with the significant changes in drug release accompanied by shape, open up new pathways to fine-tune drug release from magnetic cryogels platforms.

An additional advantage of the PVA-MNP-CA-Ac cryogel system is that it is non-fluidic, in contrast to most other hyperthermia-mediated technologies. The use of fluids is largely limited to cancer treatment, where many challenges remain. For instance, the commercial magnetic fluids available have failed to reach adequate heat dissipation potency for safe and efficient eradication of tumours^27^. Use of stronger magnetic fields can be dangerous, particularly for patients with pre-existing health conditions such as having a pacemaker or other metal implants. Moreover, insufficient uptake of fluid by the tumor as well as intracellular MNP aggregation will result in their intake by macrophages, thus hindering effectivity^60^. Unintentional distribution of fluid MNPs into excretory and other organs will lead to unwanted heat dissipation during hyperthermia treatment resulting in healthy tissue damage. The temperature control and detection achieved via magnetic hyperthermia is also limited^61^. In contrast, use of a biocompatible gel, that is stable at hyperthermia temperatures, can help address such issues and be particularly effective at sub-epidermal level hyperthermia treatment for diseases such as melanoma. Most importantly, it will allow expansion of such hyperthermia-mediated technologies into external applications such as wound care or muscle repair via external heat therapy, leading to safer, less-invasive treatment.

## Conclusions

A shape-selective magnetic cryogel system was developed as an effective platform for hyperthermia mediated, customizable drug release. The hydrogels were synthesized by first achieving a slurry comprised of 5% PVA and 5% (w/v) Fe_3_O_4_ MNPs coated with citric acid followed by hydrogen bonding with acetaminophen, where relevant. The slurries were introduced into disc and spherical cap shaped molds to create two different shapes. Gelation was achieved via freeze-thawing of the slurry-moulds at − 20/r.t. °C, 5 times, without any additional crosslinking agents.

Thermally triggered drug release behavior was shown to be significantly dependent on the shape of the cryogels. With the increase in temperature of the surrounding medium, the disc shaped gels which had twice as many loosely bound polymer edges, showed much higher levels of drug release compared to the more compactly bound spherical caps, indicating the impact of shape-mediated polymer entanglement for control of the amount of drugs released. Moreover, the discs showed effective drug release at temperatures ideal for potential *in-vivo* hyperthermia applications (42–46 °C), while the spherical caps did the same at more elevated temperatures (48–50 °C), indicating suitability for external applications such as wound care. Magnetic hyperthermia and accompanying thermal imaging experiments revealed notably different heat transfer behavior from the two shapes of gels. The drug-coated spherical caps heated much faster and were stable up to 59 °C, while the accompanying discs heated slower and retained their structural integrity up to 51 °C, demonstrating shape-dependent robustness suitable for multiple hyperthermia-mediated biomedical applications.

Overall, the amount/time of drug-release and the temperatures achieved within the cryogels were both shown to be highly tunable by changing the temperature of the medium and the shape of the gels, respectively, while keeping all other factors (Ex. gel volume, surface area, polymer/nanoparticle/rug-loading concentrations and magnetic hyperthermia conditions) constant, which has not been reported previously. These customizable features coupled with material biocompatibility and robustness, render potential for these gels to be used as an advanced hyperthermia-mediated low cost, safe and effective option for delivering drugs, with particular interest for external applications such as wound care and has potential for internal applications such as cancer treatment.

## Supplementary Information

Supplementary Information 1.

Supplementary Information 2.

## Abbreviations

PVA: Polyvinyl alcohol
MNPs: Iron (II, III) oxide magnetic nanoparticles
CA: Citric acid
Ac: Acetaminophen
DC: Direct current
EDX: Energy dispersive X-ray
UV–Vis: Ultraviolet–visible
FESEM: Field emission scanning electron microscope
FTIR: Fourier transform infrared spectroscopy
TGA: Thermo gravimetric analysis
r.t.: Room temperature
ESI: Electronic supplementary information
