# Supplementary material for: Magnetic cryogels as a shape-selective and customizable platform for hyperthermia-mediated drug delivery
Source: Sci Rep. 2022 Jun 10;12:9654. doi: 10.1038/s41598-022-13572-9 (PMC9187744; doi:10.1038/s41598-022-13572-9)
Supplement: Supplementary file 1 — Supplementary Information 1. [file 41598_2022_13572_MOESM1_ESM.docx]

Magnetic cryogels as a shape-selective and customizable platform for hyperthermia-mediated drug delivery

*Ayomi S. Perera*^1^, Richard J. Jackson^,2^, Reece M. D. Bristow^1^ and Chinyere A. White^1^*

1 Department of Chemical and Pharmaceutical Sciences, Kingston University London, Penrhyn Road, Kingston upon Thames, KT1 2EE, UK, [a.perera@kingston.ac.uk](about:blank), [k1607738@kingston.ac.uk](about:blank), [chinyere.white@gmail.com](about:blank)

2 Department of Mechanical Engineering, University College London, Torrington Place, London WC1E 7JE, UK, [r.jackson@ucl.ac.uk](about:blank)


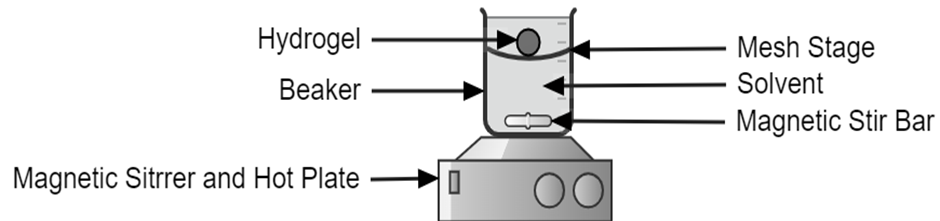


Figure S1. Schematic representation of the apparatus for the drug release studies of acetaminophen from PVA-MNP cryogels.


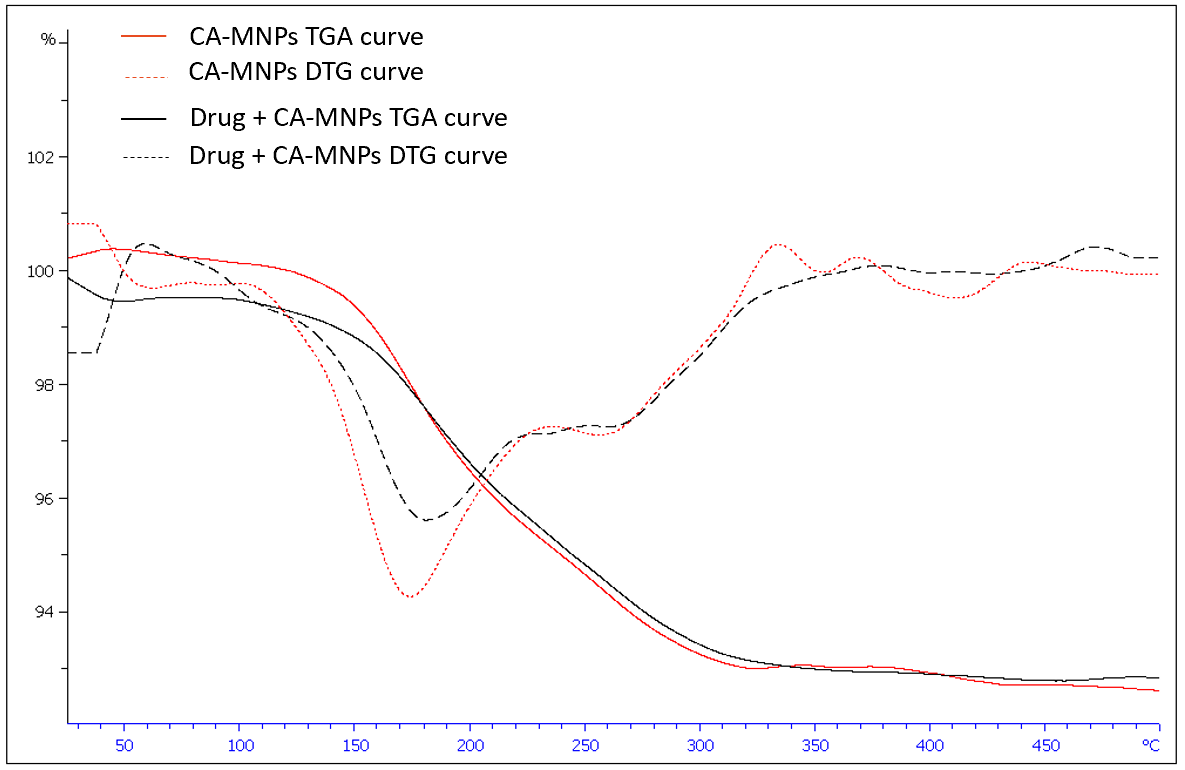


*Figure S2. The TGA and DTG curves of citric acid-coated MNPs and drug + citric acid-coated MNPs.*

Table S1. Shrinkage/expansion testing of spherical cap shaped cryogels in various mixtures of ethanol : water.

Figure S3. UV-vis absorbance scan for acetaminophen indicating a strong peak at 248 nm and a broad shoulder at 285 nm.


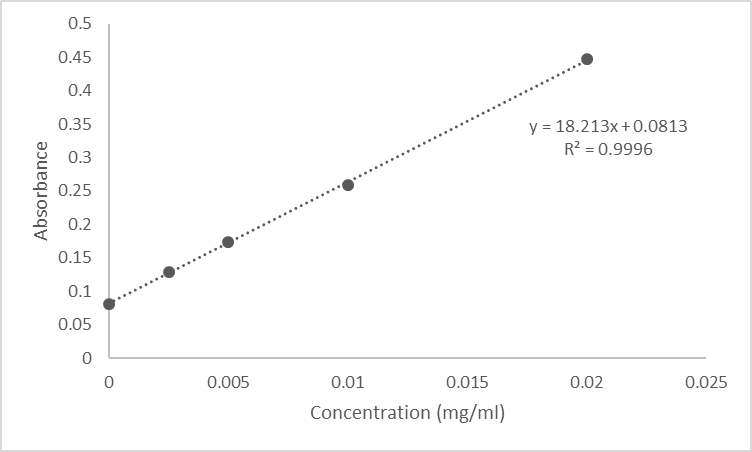


*Figure S4. The calibration curve used in the determination of the concentration of acetaminophen in IMS (25%) for the drug release studies.*

**Section 1.** Surface area calculations of disc and spherical cap gels

Disc shaped gels:

Disc radius = 1.1 cm

Disc volume = 2 ml

V_disc_ = πr^2^h

h = 0.53 cm

A_disc_ = 2 πrh + 2πr^2^ = 11.26 cm^2^

Spherical cap shaped gels

Cap radius = 1.25 cm

Cap Volume = 2 ml

V_cap_ = (πh(3r_cap_^2^ + h^2^))/6

h = 0.7 cm

A_cap_ = π(r_cap_^2^ + h^2^) + πr_cap_^2^ = 11.35 cm^2^

The difference in surface areas are <0.01% as evidenced as:

[(11.35 – 11.26) / 11.35] * 100% = 0.79%

Or

[(11.35 – 11.26) / 11.26] * 100% = 0.80%

**Section 2.** Calculation of field strength

AMF frequency f = 0.812 MHz

Field Strength from applied voltage

H = - 0.0178 V^2^ + 1.3622V – 6.2388 kA/m

For V = 19.95

fH = 11.2 x 10^9^ A/m

Safety value from literature = 5 x 10^9^ A/m ^*^

^*^Hergt R, Dutz S. Magnetic particle hyperthermia—biophysical limitations of a visionary tumour therapy. Journal of Magnetism and Magnetic Materials. 2007 Apr;311(1):187–92.


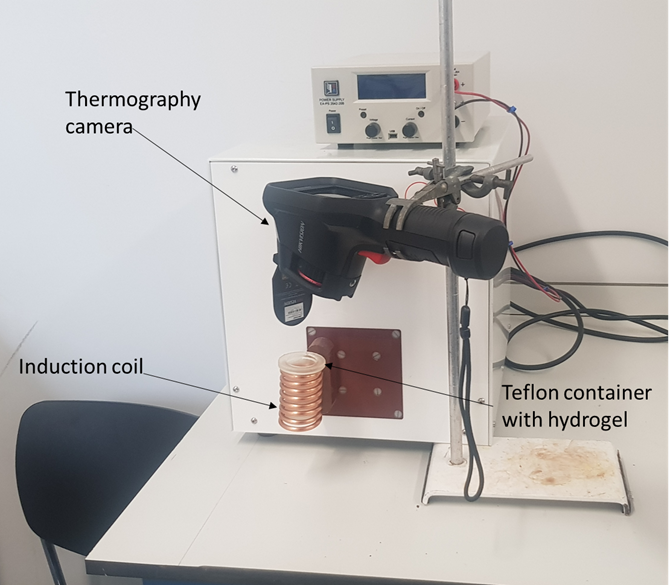


Figure S5. The assembly of the apparatus for the hyperthermia experiments of PVA-MNP cryogels.


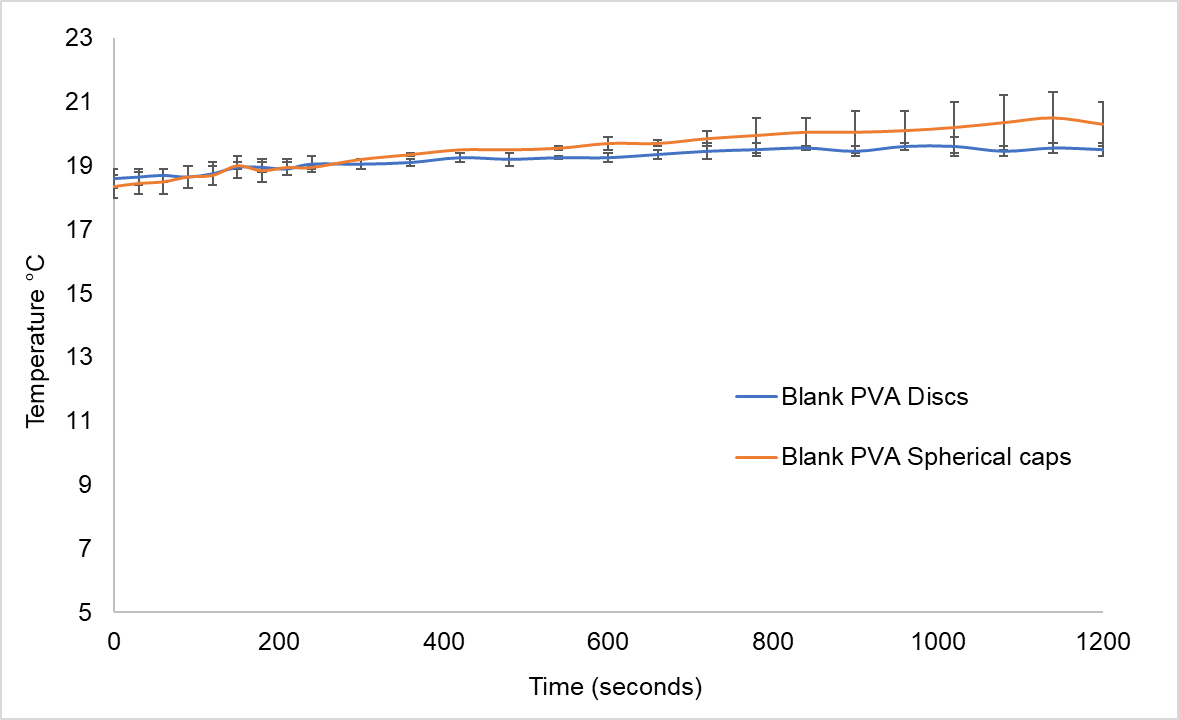


Figure S6. Temperature profile of disc and spherical cap shaped 5% PVA gels of without MNPs during magnetic hyperthermia.


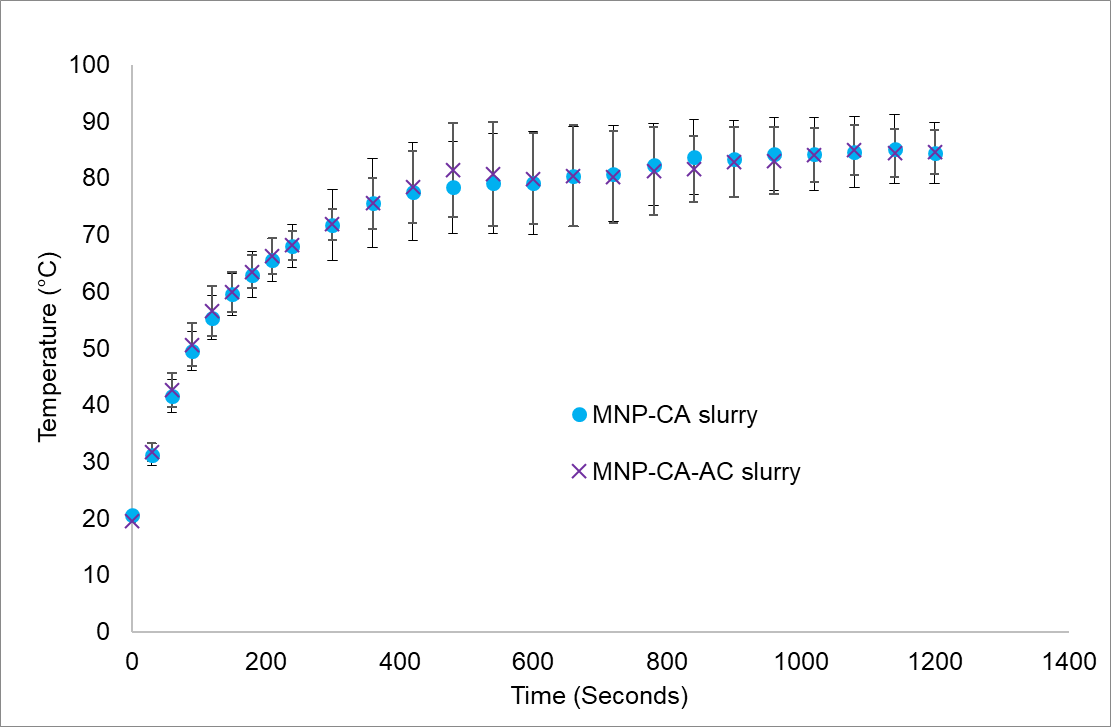


Figure S7. Comparison of temperature increase in slurries (i.e., PVA-MNP mixtures without any gel formation) with and without acetaminophen coating of MNPs during magnetic hyperthermia.
